# Cost-Effective Corrosion Detection Sensor for Above-Ground Oil and Gas Flowlines

**DOI:** 10.3390/s22218489

**Published:** 2022-11-04

**Authors:** Nader Vahdati, Oleg Shiryayev, Shahid M. Parapurath, Fook F. Yap, Haider Butt

**Affiliations:** 1 Mechanical Engineering Department, Healthcare Engineering Innovation Center (HEIC), SAN Campus, Khalifa University, Abu Dhabi 127788, United Arab Emirates; 2 Mechanical Engineering Department, 301J ECB, University of Alaska Anchorage, 3211 Providence Dr., Anchorage, AK 99508, USA; 3 Mechanical Engineering Department, Main Campus, Khalifa University, Abu Dhabi 127788, United Arab Emirates; 4 School of Mechanical and Aerospace Engineering, Nanyang Technological University, Singapore 639798, Singapore

**Keywords:** corrosion sensing, oil and gas flowlines, fiber-optic-based, fiber Bragg grating (FBG)

## Abstract

A sensor for monitoring of the external corrosion of small-diameter aboveground oil and gas pipelines (called flowlines), based on fiber-optic strain sensing, is proposed. The working principle of our proposed sensor relies on the use of a pre-stressed sacrificial structure made of the same material as the pipeline and monitoring changes in the measured strain that occur due to deterioration caused by corrosion to the structure. We present the development of analytical equations that allow designing the sensor structure to achieve the desired strain values. The analysis was verified using commercial finite element analysis (FEA) software. The proposed sensor is simple and cost-effective and can be easily manufactured. It can be deployed on existing overground pipelines without any modification to the pipeline structure. While it is not capable of measuring the corrosion rate continuously, it can provide a measurement of the average corrosion rate over the life span of its sacrificial metal structure.

## 1. Introduction

Despite the introduction of control and mitigation measures, corrosion still poses a serious threat to the integrity and safe operation of pipelines that are used for the transportation of hydrocarbons. The need for developing corrosion sensors for oil and gas flowlines (small-diameter pipelines) is ever increasing due to industry/government pipeline regulations and an aging pipeline infrastructure. According to statistics on significant pipeline incidents in the United States ranging from 2001–2020 [1], corrosion was identified as the primary cause for approximately 20% of incidents, with a total cost of approximately USD 10.9 billion in 2020. 

The oil and gas industry utilizes a variety of inspection technologies to ensure the safe operation of production and processing facilities, as well as hydrocarbon transportation infrastructure. “Pipeline Inspection Gauge” or “Pipeline Intervention Gadget” (PIG) is one of the most widely used devices for the inspection of oil and gas pipelines for deterioration due to corrosion and erosion. Long-distance transmission pipelines, due to their diameter being large and having PIG launch and retrieval stations, are regularly inspected, every 3–7 years, using PIGs. However, most oil and gas flowlines and gathering lines, found in oil fields nearer the oil wells, are not pigged, due to the unavailability of PIG launch and retrieval stations and their small diameters. This is one of the factors that creates the demand for the development of corrosion detection sensors. Another factor is that, even if flowlines and gathering lines were inspectional using PIGs, such inspections only occur once every 3–7 years. In between inspection intervals, aggressive transient pipeline corrosion may occur, which can potentially result in the release of hazardous substances such as H_2_S, explosions or fire, human fatalities, and property damage.

Large oil and gas fields may have thousands of flowlines and gathering lines, making inspections a continuous process, which requires committing a significant number of resources. A system of sensors deployed over the pipeline network will allow monitoring degradation processes and optimizing the deployment of resources by directing inspection crews to locations that are more susceptible to corrosion. In order for a sensing solution to be widely adopted by the industry, it needs to possess the following desirable characteristics [2,3]:

- It shall be non-invasive, allowing installation without modifying the pipeline structure or halting the operation of the pipeline.
- It shall be passive and not require electric power supply for operation, interrogation, or data transmission due to safety concerns in highly volatile environments.
- It shall allow obtaining near-real-time data and be able to be interrogated on demand.
- Maintenance shall be minimal, and replacement costs must be low.
- It shall be low-cost compared to existing conventional monitoring techniques.
- It shall be able to withstand the high temperature of oil and gas flowlines and gathering lines.

We believe that sensing solutions based on fiber-optic technology can satisfy most of the above-mentioned characteristics. Optical fibers are inherently safe since they do not rely on electrical currents for sensing and signal transmission. They can be deployed over large distances and possess geometric versatility due to being flexible. Fiber-optic sensors can be interrogated on demand and provide nearly real-time data readings. Finally, they may provide quasi-distributed sensing, and the cost of standard fiber is relatively low. They have already been successfully employed for the monitoring of civil infrastructure [4,5,6,7,8].

## 2. Literature Review

A large variety of corrosion sensors for application in monitoring of oil and gas infrastructure have been developed in the past several decades. A thorough review of conventional and emerging sensing technologies for corrosion monitoring is provided in [9]. In general, these sensing technologies can be divided into two types: direct sensing and indirect sensing [9]. Direct sensing technologies are capable of the monitoring and assessment of the corrosion rates. Examples of conventional direct measurement techniques include corrosion coupons, electrical resistance (ER) probes [10], and linear polarization resistance (LPR) probes [11]. Indirect corrosion measurement technologies based on fiber-optic sensors monitor various factors that affect the rate of corrosion processes (pH [12,13,14], humidity [15,16,17,18,19], temperature [17,20], concentrations of certain chemical species [21,22,23,24,25]), or their consequences, such as the presence of corrosion products [26], a reduction in wall thickness [27], changes in hoop strain [28,29,30], or leaks that occurred due to the loss of integrity [20,31]. The sensor described in this work falls into the category of indirect measurements because it does not provide the actual rate of external corrosion on the pipe wall in real-time. Instead, it provides a measurement of the corrosion rate in close vicinity to the pipe wall, which shall correlate well with the actual external corrosion rate on the exposed wall surface. 

Several sensing solutions based on optical fibers have been developed that are suitable for monitoring of external corrosion. A simple single-point sensor that allows indirect quantification of corrosion was proposed by Leung at al. [32]. The terminal end of an optical fiber is coated with a thin iron film. As the corrosion progresses, the film gets thinner; thus, less light is reflected back to the source. Simple reflectivity measurements were used to characterize corrosion process in different environments [32]. Dong et al. [33] developed a concept for a fiber-optic sensor that relies on the measurement of transmitted power through the section of the fiber with removed cladding coated with a Fe-C alloy film. As the film corrodes, more light can escape the fiber, which reduces the transmitted light power.

Tan et al. [34] developed a device based on a fiber Bragg grating (FBG) strain sensor coated with a mixture of pH-sensitive hydrogel and polydimethylsiloxane (PDMS). Hydrogels represent cross-linked polymers that are highly hydrophilic and, therefore, can absorb water and swell due to the change in volume. The expansion and contraction of the coating layer induces strain in the fiber, which can be detected by interrogating the FBG strain sensor. A similar principle was utilized in the work by Hu et al. [35], who developed a metallized (Fe-C coated) FBG sensor, where the corrosion products induce strain due to the fact that they occupy a larger volume. 

Chen at al. [36,37] developed a sensor based on long-period fiber gratings (LPFGs) for measuring corrosion in carbon steel. LPFGs are similar to FBGs, but with much longer periods, which allows coupling the light from the core mode into the cladding modes, creating attenuation bands in the transmission spectra. Interactions of light in the cladding modes and changes in the effective refractive indices of the cladding allow using these sensors for a variety of practical purposes [38]. The study presented in [36,37] showed a correlation between the mass loss of Fe-C coating deposited on LPFG with changes in the transmission spectrum. An improved version of an earlier prototype that was enhanced by the utilization of graphene and a silver nanowire film composite for electroplating of a Fe-C alloy was reported recently in [39]. A similar study with a pure Fe coating was reported by Coelho et al. [40]. 

Another concept that allows monitoring of external corrosion is to use pre-stressed steel structures that are exposed to the corrosive environment and monitor the change in induced strain as the corrosion process progresses, causing a reduction of the structure’s stiffness due to the loss of material [41].

In [42], a fiber-optic-based corrosion detection sensor consisting of a semi-circular plastic curved beam attached to a dog-bone-shaped metal component, with the exact same material properties as the pipeline, was described. The complex design of the dog-bone-shaped metal made the design difficult to manufacture and costly, thus impractical for use in the actual oil field. In addition, since the semi-circular component of [42] was made of plastic, permanent set and drift were of concern, particularly at high temperatures. 

It is important to mention that, in most oil fields, three to four thousand flowlines and gathering lines with a length of 1 km to 4 km may be found. On average, there may be 3500 flowlines/gathering lines with an average length of 2.5 km. If corrosion monitoring is performed every 5 m, at least 1.75 million corrosion sensors (500 sensors per pipeline times 3500) would be needed to monitor the corrosion health of flowlines in just one oil field. Since many sensors may be needed, there is a well-founded need to keep the cost of the corrosion detection sensor very low. In addition to the sensor cost, there will be costs due to optical fibers, interrogator(s), installation, and possibly, optical switches. The crucial key is to keep the cost of the corrosion sensor down, since there are too many of them.

In this paper, a cost-effective fiber-optic based corrosion detection sensor based on corrosion-induced strain change is proposed for external corrosion detection of oil and gas pipelines. Compared to the concept described in [41], the strain here is measured on the non-corroding elastic structure, improving reliability and robustness. 

Two alternative corrosion detection sensor concepts, which are easier to manufacture and use than the sensor of [42], and thus less expensive, are discussed. Design concept #2, in particular, is the focus, where the semi-circular plastic component is replaced with a U-shaped metal component, which is very easy to manufacture and eliminates the concern of permanent set and drift. The dog-bone-shaped component was simplified and replaced with a U-shaped metal, made from the same pipeline material. Even though the sensor design of this paper has a similar working principle as the sensor of [42], the design equations are completely different. In this paper, the equations to design the new corrosion detection sensors are given. In addition, the effect of temperature on the corrosion sensor is also discussed, which was not covered in [42].

The geometry of the sensor was analyzed using Castigliano’s second theorem, and the analysis results were verified using the FEA method. The proposed sensor was manufactured, and its performance was evaluated using the accelerated corrosion test. Excellent correlation among the theory, FEA, and experimental data was observed. The experimental results demonstrated the feasibility of the proposed sensor. The only obstacle is that the proposed corrosion detection sensor of this research is unable to measure corrosion rate continuously, but it can provide the average corrosion rate over the life span of its sacrificial metal.

## 3. Corrosion Detection Sensor Design

The corrosion detection sensor of this paper will be placed on the outer surface of oil and gas pipelines to detect pipeline external corrosion (see Section 7.1). In this paper, the focus is on 2- to 12-inch pipelines called flowlines and gathering lines, which are connected to the wellhead or connecting several wells to the manifold. Flowlines, since they are nearest the oil wells, can be as hot as 150 to 315 degrees Celsius, depending on the oil field. Reference [43] indicated that high-pressure high-temperature (HPHT) reservoirs start at bottomhole pressures of 69 MPa (10,000 psi) and temperatures of 150 °C (300 F). In some oil reservoirs, the bottomhole pressures and temperatures can be as high as 275 MPa (40,000 psi) and 315 °C (600 F), although there are flowlines that can operate at temperatures lower than 150 °C.

The flowlines that are the focus of this paper have a temperature of 130 °C; so, the material used to make the corrosion detection sensor for this study needs to be able to withstand temperatures as high as 130 degrees Celsius.

In this paper, two corrosion detection sensor concepts are shown. The equations to design the two proposed sensors are given in this paper, and the feasibility of using the sensor on flowlines is also discussed.

### 3.1. Corrosion Detection Sensor—Design Concept #1

**Figure 1:**
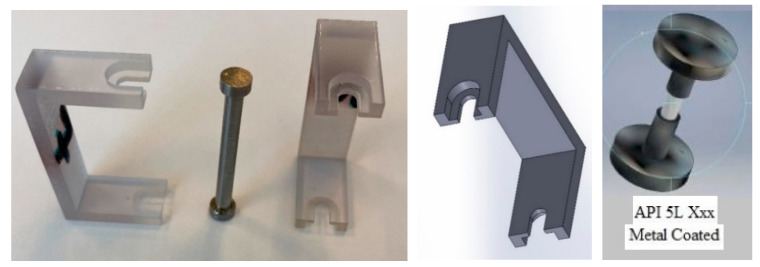
The 3D-printed external corrosion detection sensor and its CAD model.

The sensor of Figure 1 consists of a 3D-printed or molded U-shaped plastic part (shown in transparent color in Figure 1), a dumbbell-shaped sacrificial metal made from the same material as the pipeline, and an optical fiber with FBG sensors. 

**Figure 2:**
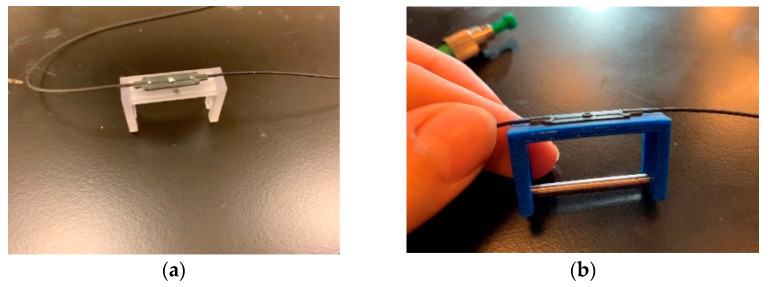
U-shaped corrosion sensor with an FBG sensor bonded to its upper surface. (**a**) W/O dumbbell-shaped component. (**b**) With dumbbell-shaped component.

In this design, the transparent plastic part, made of high-temperature plastic, is permanently glued to each FBG sensor, as shown in Figure 2. Figure 2 shows the U-shaped plastic component with an FBG sensor bonded to its the upper surface. When the dumbbell-shaped metal component, made from the same pipeline material, is inserted into the U-shaped plastic component, the plastic component deforms (see Figure 2b) and, thus, creates tension at the FBG sensor location; thus, a signal will be observed at each FBG sensor location on the interrogator display.

To prevent the dumbbell-shaped metal from crevice corrosion, the ends of the dumbbell-shaped metal were dipped in liquid plastic or coated with an epoxy, as shown in Figure 1. Coating the ends forces the corrosion to only take place at the center of the dumbbell-shaped metal, thus eliminating or reducing crevice corrosion.

It is important to mention that, in the sensor design of Figure 1 and Figure 2, since the U-shaped plastic component can be in a deformed state for an extended period, the possibility of facing high temperatures (150 °C to 315 °C in the case of oil flowlines) and environmental temperature and humidity conditions (extreme dessert temperature and humidity conditions in the case of oil flowlines), a low creep and permanent set plastic material should be chosen. In addition, the plastic material needs to be insensitive to ozone, and its mechanical properties should remain relatively constant with aging. Polyether ether ketone (PEEK) plastic maintains stiffness up to 170 °C and melts at 343 °C, and it has an outstanding resistance to harsh chemicals. Some grades of PEEK have a useful operating temperature of up to 250 °C. In addition, PEEK has excellent creep resistance at room temperature, but at temperatures beyond 130 °C, which is the case for our application, the creep goes up. Torlon (polyamide-imide) plastic has a maximum service temperature of 200 to 220 °C, and among all engineering plastics, it has the lowest coefficient of thermal expansion and very low creep. Therefore, it can be a great candidate material for design concept #1. To be safe, the authors of this paper recommend sensor design concept #1 for flowlines that operate below 130 °C.

### 3.2. Corrosion Detection Sensor—Design Concept #2

**Figure 3:**
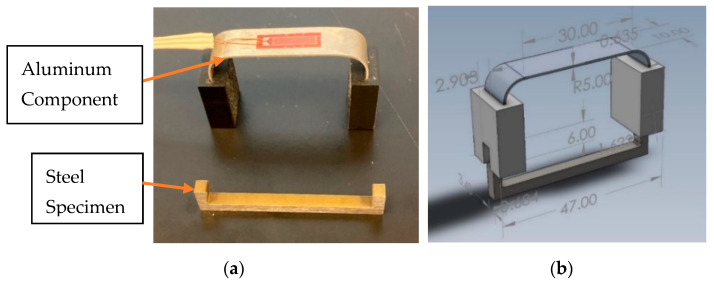
(**a**) Actual corrosion sensor; (**b**) corrosion sensor CAD model.

The sensor, shown in Figure 3, consists of an aluminum U-shaped component, two identical 3D-printed plastic components, shown in black color in Figure 3a, and a sacrificial steel specimen (again U-shaped) made from the same material as the oil or gas pipeline under corrosion investigation. The strain of the aluminum U-shaped component can be measured using a strain gage (as shown in Figure 3a) or an optical fiber with an FBG sensor attached to the top surface of the aluminum component. Figure 3b shows the assembled 3D CAD model and the sensor dimensions in mm.

**Figure 4:**
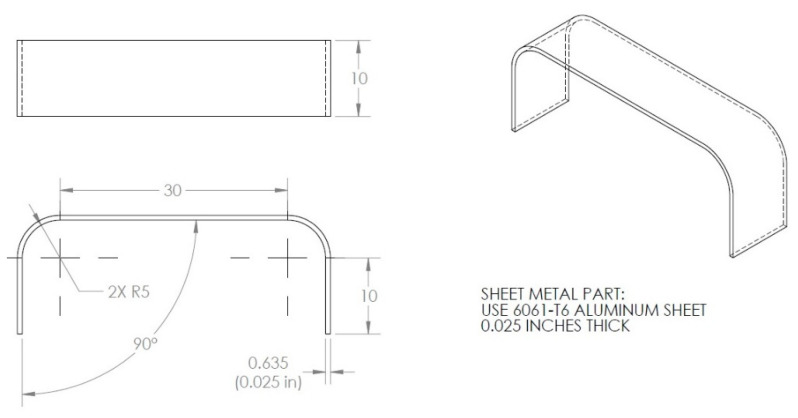
Aluminum U-shaped sheet metal (dimensions in mm).

**Figure 5:**
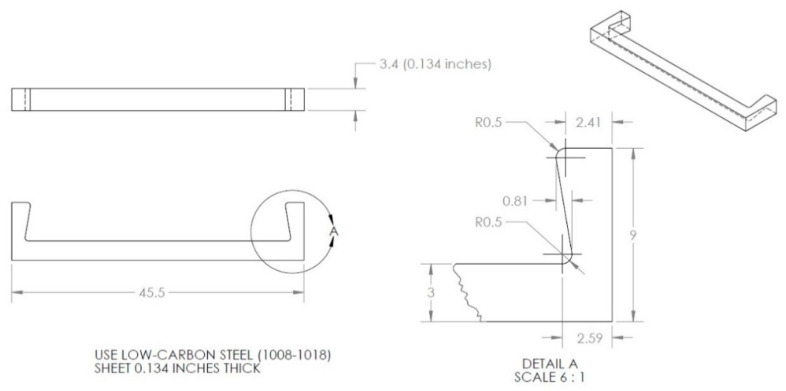
Sacrificial steel specimen, made from the same oil and gas pipeline material.

**Figure 6:**
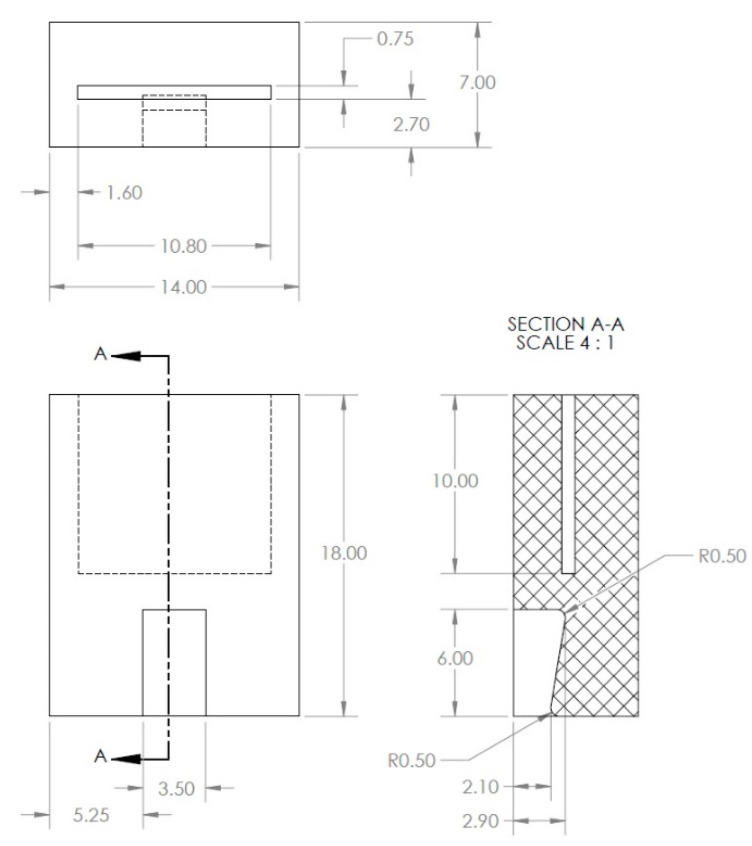
Plastic component dimensions.

The exact dimensions of the corrosion detection sensor components, used in our experiments, are given in Figure 4, Figure 5 and Figure 6. The aluminum U-shaped component, shown in Figure 4, is 0.635 mm thick and 10 mm wide and made of AL 6061-T6 material.

The dimensions of the U-shaped sacrificial steel component, made from the same material oil and gas pipeline, is shown below. The steel specimen of Figure 5 was machined from the API-5L-X65 material, commonly used for oil and gas pipelines.

The black-colored plastic components, shown in Figure 3a, have the following dimensions as shown in Figure 6. As explained earlier, since the flowlines and gathering lines (2- to 12-inch pipelines) can be as hot as 150 to 315 degrees Celsius (in our application, 130 °C), the material used to make the black plastic components should be a high-temperature plastic. For example, VISIJET M2S-HT250, from 3D systems, can withstand temperatures as high as 250 degrees Celsius, or PEEK or Torlon can be good candidate materials for this component. This component does not have to be plastic, and it can also be made from metal.

**Figure 7:**
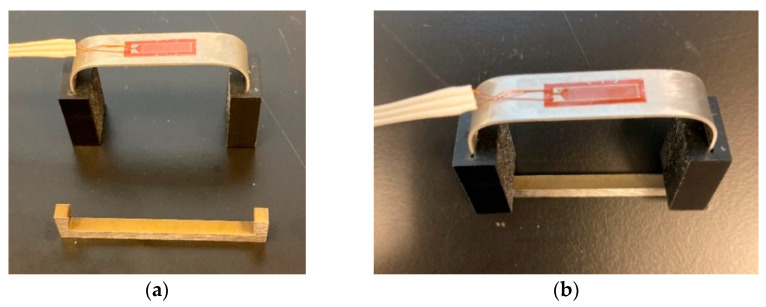
(**a**) Steel specimen before assembly (**b**) and after assembly.

When the U-shaped sacrificial steel specimen (see Figure 5) is inserted into the two black-colored plastic pieces, it places the steel specimen and the top surface of the aluminum U-shaped component in tension. When the steel specimen corrodes and breaks into two pieces, the tension on the top surface of the aluminum U-shaped component is relieved. The sensor of Figure 7 acts very similarly to the sensor design of Figure 1, expect it is made of aluminum.

In this paper, two corrosion detection sensor designs are proposed, and both designs can be used depending on the application and the pipeline operating temperatures. We recommend design #1 for low-temperature applications and design #2 for high-temperature applications. Since the focus of this paper is on oil flowlines and the operating temperature for our application is near 130 °C, the 2nd corrosion detection sensor design is the focus, since it can withstand higher temperatures and the sensor creep should be very low.

Please refer to Section 7.1 “Field Implementation” to see the authors’ vision of how to use this paper’s proposed corrosion detection sensors in the field.

### 3.3. Sensor Fabrication

**Figure 8:**
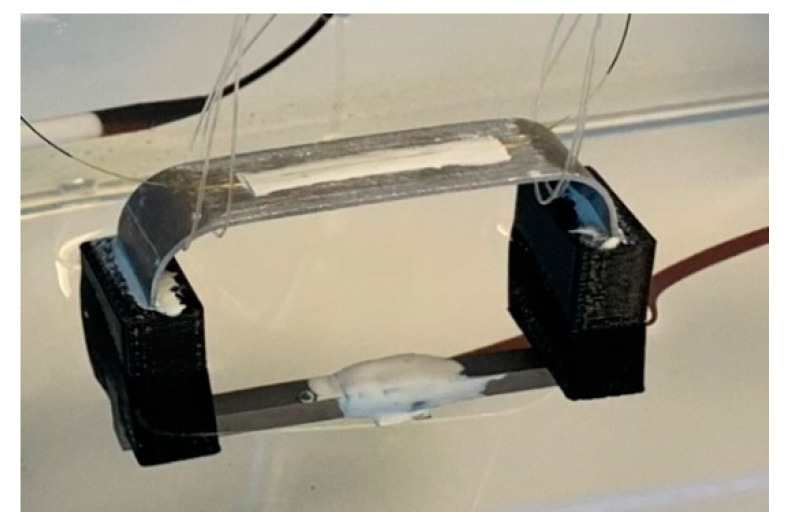
Corrosion sensor with an optical fiber with many FBG sensors.

**Figure 9:**
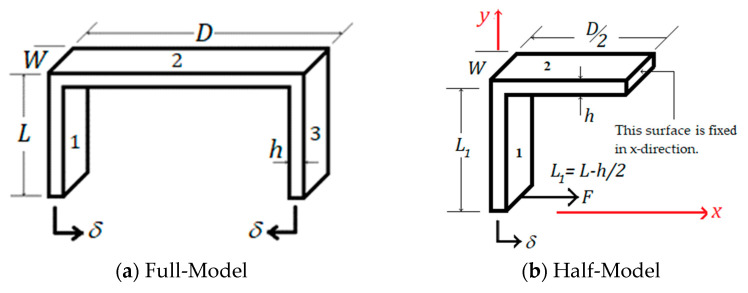
Plastic or aluminum component subject to displacement *δ* at the free ends.

To fabricate the sensor design of Figure 3, metal stamping was used to build the aluminum U-shaped component (6061-T6 aluminum). There are many manufacturers that can stamp aluminum, structural steels, and stainless-steel materials with thicknesses between 0.5 and 15.8 mm (0.020–0.625″). The 6061-T6 aluminum sheet, used in this study, was only 0.635 mm thick; so, it was not an issue to form the design of Figure 8 using stamping, but during the manufacturing of this component, great attention must be given to the 90-degree angle and the 5 mm fillet radii, shown in Figure 4. If the 90-degree angle is not maintained, the strain obtained at the top surface of the aluminum U-shaped part will not be the same strain that the sensor was designed for. If the 90-degree angle is just off by 1 degree (0.0175 radians), the displacement *δ* given to both ends of the aluminum U-shaped part (see Figure 9a) can be off by ±0.3 mm.

The two black-colored plastic components were 3D-printed and were inserted into the two ends of the aluminum U-shaped component. An optical fiber with an FBG sensor (with a strain limit of 5000 με), shown underneath the white-colored glue, was bonded to the top surface of the aluminum U-shaped component, as shown in Figure 8.

## 4. Design Equations for Sensor Design Concepts #1 and #2

### 4.1. Plastic or Aluminum U-Shaped Component Strain

To be able to design the corrosion sensor concepts #1 and #2 at different sizes, we needed to develop closed-form design expressions for the above proposed sensors. Most optical fibers can measure strain up to a limit. Design expressions are therefore necessary to make sure the strain of the optical fiber at the FBG sensor locations does not exceed the manufacturer-recommended strain limit, or else, the fiber will crack and break. Different tensile strengths have been reported for single-mode optical fibers, and the strain limit is generally between 5000 με and 10,000 με.

In this section, closed-form expressions for the strain of the plastic or aluminum U-shaped component (see Figure 2a and Figure 3a) are developed and compared with the FEA strain results. Buckling/bending and pipeline temperature effects on strain were ignored. The U-shaped component consists of three beams, two vertical beams (labeled as 1 and 3) and one horizontal beam (labeled as 2); see Figure 9a.

**Figure 10:**
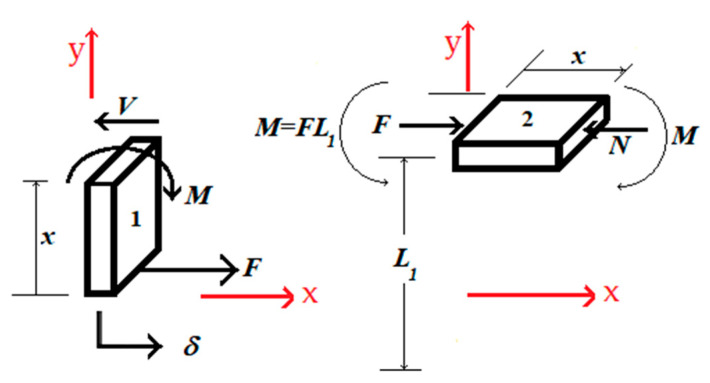
Internal shear forces and bending moments.

To find a closed-form equation for strain on the top surface of beam 2 (where the FBG sensor is attached), we used the half-beam model of Figure 9b. The vertical beam (labeled as 1 in Figure 10), when given a force *F*, as shown, will develop the shear force *V* and bending moment *M* at distance *x,* given by Equations (1) and (2) shown below.

(1) V=F

(2) M=Fx

The strain energy developed in beam 1 is equal to:

(3) $$U_{1}=\int_{0}^{L_{1}}\frac{M^{2}}{2EI}dx+\int_{0}^{L_{1}}\frac{CV^{2}}{2GA}dx$$

where *E* is the Young’s modulus of the plastic or the aluminum U-shaped component, *I* is the area moment of inertia, *G* is the shear modulus, *A* is the cross-sectional area, and *L*_1_ is the distance from the point where the force *F* is applied to the center of beam 2. The area moment of inertia, *I*, for beam 1 is equal to $I=\frac{1}{12}wh^{3}$. The parameter *C* in the 2nd term of Equation (3) is the strain energy correction factor for transverse shear, equal to 1.2 when the cross-section is rectangular. For beam 2, we can write the following strain energy function:

(4) $$U_{2}=\int_{0}^{\frac{D}{2}}\frac{M^{2}}{2EI}dx+\int_{0}^{\frac{D}{2}}\frac{N^{2}}{2EA}dx$$

where the normal force *N* and the bending moment *M* are equal to:

(5) $$N=F\;\mathrm{and}\;M=FL_{1}$$

The total strain energy, for beam 1 and half of beam 2, is equal to $U=U_{1}+U_{2}$:

(6) $$U=\int_{0}^{L_{1}}\frac{{\left(Fx\right)}^{2}}{2EI}dx+\int_{0}^{L_{1}}\frac{CF^{2}}{2GA}dx+\int_{0}^{\frac{D}{2}}\frac{{\left(FL_{1}\right)}^{2}}{2EI}dx+\int_{0}^{\frac{D}{2}}\frac{F^{2}}{2EA}dx$$

The deflection *δ*, at the location where the force *F* is applied is equal to:

(7) $$\delta =\frac{dU}{dF}=\frac{F}{EI}\left(\frac{L_{1}^{3}}{3}\right)+\frac{CF}{GA}L_{1}+\frac{FL_{1}^{2}}{EI}\left(\frac{D}{2}\right)+\frac{F}{EA}\left(\frac{D}{2}\right)$$

and since E=2(1+μ)G or $G=\frac{E}{2\left(1+\mu \right)}$, then we obtain:

(8) $$\delta =\frac{dU}{dF}=\frac{FL_{1}^{3}}{3EI}+\frac{2\left(1+\mu \right)CFL_{1}}{EA}+\frac{FL_{1}^{2}D}{2EI}+\frac{FD}{2EA}$$

In our application, when the dumbbell metal or the sacrificial steel specimen is inserted into the U-shaped plastic or aluminum part, we imposed a deflection *δ* at the edges. Knowing the deflection *δ*, then the force *F* can be calculated using Equation (9):

(9) $$F=\frac{\delta }{\frac{1}{E}\left(\frac{L_{1}^{3}}{3I}+\frac{2\left(1+\mu \right)CL_{1}}{A}+\frac{L_{1}^{2}D}{2I}+\frac{D}{2A}\right)}$$

and the tension stress, $\sigma_{t}$, experienced by beam 2, at its top surface, is equal to:

(10) $$\sigma_{t}=\frac{MC}{I}-\frac{N}{A}=\frac{FL_{1}\frac{h}{2}}{\frac{1}{12}wh^{3}}-\frac{F}{wh}$$

(11) $$\sigma_{t}=\left(\frac{6L_{1}}{wh^{2}}-\frac{1}{wh}\right)F=\left(6L_{1}-h\right)\frac{F}{wh^{2}}$$

Knowing the tension stress, the tension strain experienced by the top side of beam 2 and the FBG sensor will be equal to:

(12) $$\epsilon_{t}=\left(6L_{1}-h\right)\frac{F}{Ewh^{2}}$$

In Equation (12), substituting for force *F* with Equation (9), we obtain Equation (13):

(13) $$\epsilon_{t}=\frac{\delta \left(6L_{1}-h\right)}{wh^{2}\left(\frac{L_{1}^{3}}{3I}+\frac{2\left(1+\mu \right)CL_{1}}{A}+\frac{L_{1}^{2}D}{2I}+\frac{D}{2A}\right)}$$

As can be seen from Equation (13), the strain is not a function of Young’s modulus, *E*, but a weak function of Poisson’s ratio, implying that the material of the U-shaped plastic or the aluminum component plays little role in the top surface strain of beam 2. Basically, the strain $\epsilon_{t}$ is a function of the U-shaped component dimensions and the applied displacement *δ*.

### 4.2. Buckling and Bending Effect on Strain

**Figure 11:**
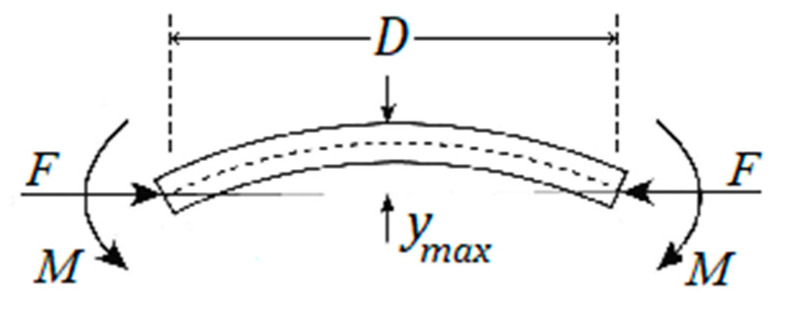
Forces and moments experienced by beam 2.

Beam 2, shown in Figure 9a, experiences the following forces and bending moments; see Figure 11.

As a result of these forces and moments, beam 2 can possibly buckle, if one is not careful. Referring to [44], the critical buckling load is given below:

(14) $$F_{cr}=\frac{\pi^{2}EI}{D^{2}}$$

To avoid buckling, force *F* should be kept below $F_{cr}$. In Equation (14), *E* is Young’s modulus, *I* the area moment of inertia, and *D* the length of beam 2. The maximum deflection, $y_{max}$, occurs at *D*/2, and it is equal to:

(15) $$y_{max}=L_{1}\left(Sec\left(\frac{\pi }{2}\sqrt{\frac{F}{F_{cr}}}\right)-1\right)$$

Accounting for extra stress at the top surface of beam 2, due to bending, Equation (10) is modified as follows:

(16) $$\sigma_{t}=\frac{M_{max}C}{I}-\frac{N}{A}$$

where $M_{max}$ is equal to:

(17) $$M_{max}=FL_{1}+Fy_{max}$$

The tension stress, $\sigma_{t}$, experienced by beam 2, at its top surface, accounting for bending, is now equal to:

(18) $$\sigma_{t}=\frac{M_{max}C}{I}-\frac{N}{A}=\frac{Fy_{max\;}C}{I}+\frac{FL_{1}C}{I}-\frac{F}{wh}$$

or simplified to:

(19) $$\sigma_{t}=\left(6y_{max}+6L_{1}-h\right)\frac{F}{wh^{2}}$$

The revised strain at the top surface of beam 2 is equal to:

(20) $$\epsilon_{t}=\frac{\delta \left(6y_{max}+6L_{1}-h\right)}{wh^{2}\left(\frac{L_{1}^{3}}{3I}+\frac{2\left(1+\mu \right)CL_{1}}{A}+\frac{L_{1}^{2}D}{2I}+\frac{D}{2A}\right)}$$

To verify Equations (13) and (20), a comparison between Equations (13) and (20) was made against the finite element (FE) results. It is important to mention that design Equations (13) and (20), provided in this paper, are not meant to provide precise strain values on the top surface of the U-shaped part, but these equations are provided to make sure the optical fiber strains are kept below the strain limit of the fiber. Since all the sensor components have dimensional tolerances, there is shrinkage during the 3D printing of the plastic components, and due to other environmental factors that can affect sensor dimensions and angles, the actual strain experienced by the fiber and the U-shaped part may be different from the designed strain; however, as long as the strain of the optical fiber is below its strain limit, the corrosion detection sensor will work just fine.

### 4.3. Pipeline Temperature Effect on Strain

To use the corrosion detection sensor of this paper, it is important that the oil and gas pipeline operators account for the impact of pipeline temperature and daily pipeline temperature fluctuations on the corrosion sensor strain. The temperature and internal pressure of the flowlines, being very near the oil wells, can be very high, depending on the oil reservoir. As explained earlier, the surface temperature of oil and gas flowlines can be anywhere from 150 °C to 315 °C (in our case, 130 °C), depending on the oil reservoir.

Pipeline temperature fluctuation should be also considered when designing the corrosion sensor. The pipeline temperature fluctuation depends on how continuous the oil production is. Most oil fields run 24 h a day, 365 days a year, and the pipeline temperature fluctuations are minimal. However, if there are frequent showdowns and startups, the pipeline temperature fluctuation can be large; thus, the fluctuation in the corrosion sensor strain can be large.

The best way to know the actual surface temperature of the pipeline and the pipeline temperature fluctuation, at locations where the corrosion detection sensors sit, is to run a fiber-optic temperature sensor along with the bundle of optical fibers used to measure the strain of the corrosion detection sensors. In this section, with the focus being on sensor design concept #2, the effect of pipeline temperature on sensor strain is investigated. 

As was explained earlier, the length of the sacrificial steel specimen is smaller than the length of the aluminum part/plastic part assembly. Therefore, when the sacrificial steel specimen is placed on the aluminum part/plastic part assembly, the aluminum component bends, and the top surface of the aluminum component goes into tension. The tension strain created on the top surface of the aluminum component due to the sacrificial steel specimen is referred to as the mechanical strain, $\epsilon_{m}$.

When the corrosion detection sensor of this paper comes in contact with the hot oil and gas pipeline, the temperature of the corrosion detection sensor will naturally rise and eventually reach the same temperature as the pipeline. Since the optical fiber is bonded to the U-shaped aluminum component, it will naturally experience mechanical strain, induced by the sacrificial steel specimen on the aluminum part/plastic part assembly, plus the strain due to the pipeline temperature change. The strain due to the pipeline temperature change consists of two strains, which will be explained later.

For a bare optical fiber with an FBG sensor, the mechanical strain and the temperature change cause a shift in the reflected wavelength of the FBG, as shown below.

(21) $$\frac{\Delta \lambda }{\lambda_{B}}=\left(1-p_{e}\right)\epsilon_{m}+\left(\alpha_{f}+\xi \right)\Delta T$$

where $\lambda_{B}$ is the central Bragg wavelength, Δλ is the wavelength shift, $p_{e}$ is the photo-elastic coefficient (0.22 for a bare optical fiber made of SiO_2_), $\epsilon_{m}$ is the mechanical strain given to the FBG, $\alpha_{f}$ is the thermal expansion coefficient of the fiber, ξ is the thermo-optic coefficient, and ΔT is the thermal gradient. When the optical fiber with an FBG sensor is bonded to a host material, in our case the aluminum component, then a modified formula should be used as follows:

(22) $$\frac{\Delta \lambda }{\lambda_{B}}=\left(1-p_{e}\right)\epsilon_{m}+\left[\left(\alpha_{f}+\xi \right)+\left(1-p_{e}\right)\left(\alpha_{h}-\alpha_{f}\right)\right]\Delta T$$

where $\alpha_{h}$ is the coefficient of thermal expansion of the host material. The equation above clearly indicates that there will be a shift in the Bragg wavelength due to both mechanical strain and temperature change.

As explained in Section 4.1 and Section 4.2, when the sacrificial steel specimen is attached to the U-shaped aluminum/plastic assembly at room temperature, the top surface of the aluminum component will experience the mechanical tension strain given below (see Equation (20)).

$$\epsilon_{m}=\epsilon_{t}=\frac{\delta_{m}\left(6y_{max}+6L_{1}-h\right)}{wh^{2}\left(\frac{L_{1}^{3}}{3I}+\frac{2\left(1+\mu \right)CL_{1}}{A}+\frac{L_{1}^{2}D}{2I}+\frac{D}{2A}\right)}$$

where $\delta_{m}$ is the mechanical deflection created by the sacrificial steel specimen being attached to the aluminum part/plastic part assembly. As explained in Figure 9a, *δ* is the deflection given to each end of the aluminum part/plastic part assembly.

When the sensor temperature is above room temperature, additional deflection will be induced on the aluminum component by the temperature change due to the fact that aluminum, plastic, and steel have different coefficients of thermal expansions. Let us refer to that additional deflection due to the temperature change and the coefficient of thermal expansion mismatch as $\delta_{\Delta T-\alpha }$.

When both the mechanical deflection and Δ*T* are present, $\delta_{m}$ in the above equation should be replaced by the following equation:

(23) $$\delta_{total}=\delta_{m}+\delta_{\Delta T-\alpha }$$

Since the optical fiber with an FBG sensor is attached to the top surface of the U-shaped aluminum component, the optical fiber will experience additional strain due to the temperature change of the aluminum component; thus, the total strain experienced by the FBG sensor will be equal to:

(24) $$\epsilon_{total}=\frac{(\delta_{m}+\delta_{\Delta T-\alpha })\left(6y_{max}+6L_{1}-h\right)}{wh^{2}\left(\frac{L_{1}^{3}}{3I}+\frac{2\left(1+\mu \right)CL_{1}}{A}+\frac{L_{1}^{2}D}{2I}+\frac{D}{2A}\right)}+(\alpha_{Al}-\alpha_{f})\Delta T$$

The thermal expansion coefficient of silica is 0.41 × 10^−6^ C^−1^. The thermal expansion coefficient of aluminum is 24 × 10^−6^ C^−1^ (58.5-times greater than the optical fiber, made of silica). Therefore, we can eliminate the coefficient of thermal expansion of the fiber in Equation (24). If we expand Equation (24), with $\alpha_{f}$ ignored, we obtain:

(25) $$\epsilon_{total}=\frac{\delta_{m}\left(6y_{max}+6L_{1}-h\right)}{wh^{2}\left(\frac{L_{1}^{3}}{3I}+\frac{2\left(1+\mu \right)CL_{1}}{A}+\frac{L_{1}^{2}D}{2I}+\frac{D}{2A}\right)}+\frac{\delta_{\Delta T-\alpha }\left(6y_{max}+6L_{1}-h\right)}{wh^{2}\left(\frac{L_{1}^{3}}{3I}+\frac{2\left(1+\mu \right)CL_{1}}{A}+\frac{L_{1}^{2}D}{2I}+\frac{D}{2A}\right)}+\alpha_{Al}\Delta T$$

where $\delta_{\Delta T-\alpha }$ can be found as follows:

(26) $$\delta_{\Delta T-\alpha }=\delta_{Al}+\delta_{Pl}-\delta_{St}$$

All the terms of Equation (26) will be defined in more detail later in Section 6.2. Please notice that Equation (25) includes the strains of Section 4.1, Section 4.2 (term #1), and Section 4.3 (terms #2 and #3), all in one equation. To verify Equation (25), in particular the last two terms of Equation (25), an experiment was conducted, and the results of that experiment can be found in Section 6.2.

## 5. Numerical Validation of Design Equations Using FEM (W/O the Temperature Effect)

**Figure 12:**
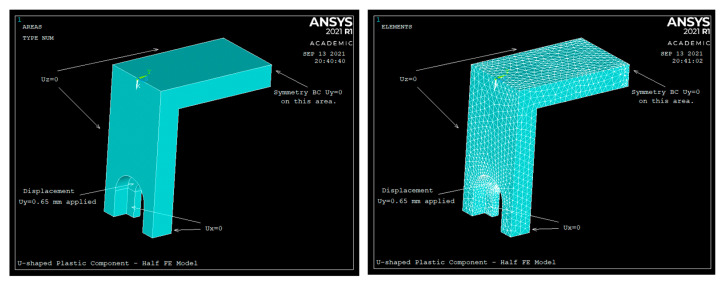
Half ANSYS FE model and its boundary conditions.

To validate Equations (13) and (20), an FE model of the U-shaped component was developed in ANSYS. Due to symmetry, only half of the U-shaped component was modeled in ANSYS. To validate Equations (13) and (20), the sensor of Figure 2, made of plastic, was used, with the dimensions shown in Table 1. All the material properties are also shown in Table 1. SOLID187 10-noded tetrahedral elements were used to mesh the CAD model. The FE models, and the boundary conditions given to the ANSYS FE model, are shown in Figure 12.

To verify Equations (13) and (20) against the ANSYS results, the U-shaped plastic component was given a displacement of *δ* = 0.65 mm at a distance *L*_1_; see Table 1. The rows highlighted by the light gray color are the outputs.

**Figure 13:**
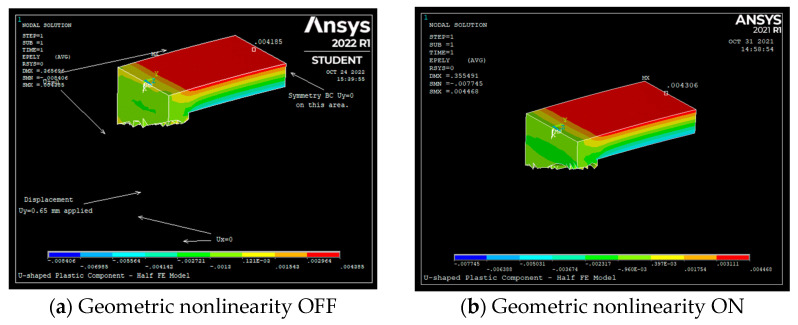
Tension strain (*ε_y_*) observed in ANSYS on the top surface of beam 2.

Figure 13 shows the tension strain (*ε_y_*) observed in ANSYS on the top surface of beam 2 with geometric nonlinearity turned OFF (Geom OFF). Figure 13 indicates that the strain at the middle of the top surface of beam 2 is 0.004185 (4185 με). When the geometric nonlinearity is OFF, Table 1 clearly shows that the strain calculated by Equation (13) is only 3.6% off from the ANSYS strain results. When the geometric nonlinearity is OFF in ANSYS, buckling/bending effects are basically ignored.

To verify Equation (20), meaning to account for the effect of buckling/bending on the tension strain of the top surface of the U-shaped plastic component, the geometric nonlinearity was turned ON in ANSYS. Figure 13b indicates that the strain at the middle of the top surface of beam 2 is 0.004306 (4306 με). When the geometric nonlinearity is ON, Table 1 indicates that the strain calculated by Equation (20) is 3.1% off from the ANSYS strain results. The ANSYS tension strain results correlate very well with the analytical results.

## 6. Experimental Validation

### 6.1. Experimental Validation of Theory at Room Temperature and Accelerated Corrosion Test

**Figure 14:**
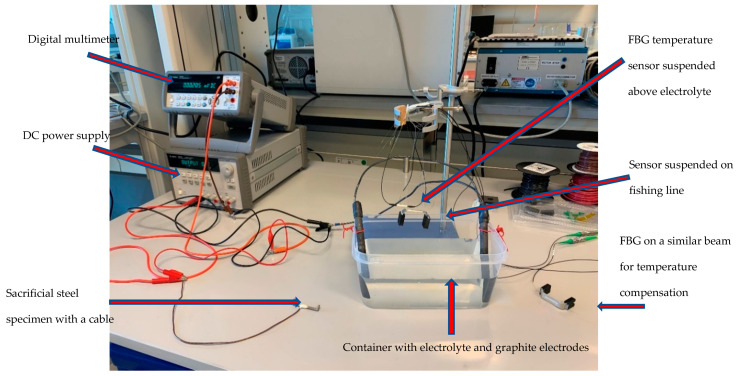
Experimental setup prior to inserting the sacrificial steel part.

The sensor prototype of Figure 8 was experimentally tested in an accelerated corrosion test to verify the viability of the proposed corrosion sensor design and demonstrate the working principle of the sensor in an actual corrosive environment. In this study, an electrochemical cell was employed to accelerate corrosion in the fabricated corrosion detection sensor to shorten the experimental time. The accelerated corrosion test is shown in Figure 14.

**Figure 15:**
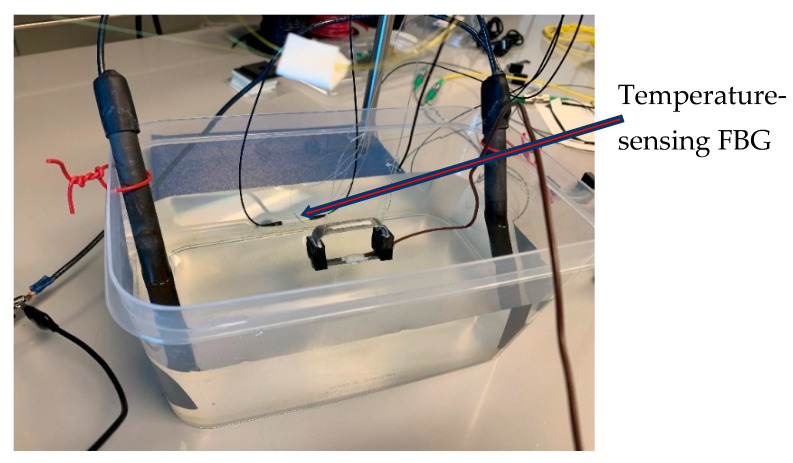
Experimental setup after inserting the sacrificial steel part.

The experimental setup consisted of an electrolyte solution (a 3.5% by weight NaCl solution), a plastic tank, two graphite electrodes, two corrosion detection sensors, each with an FBG sensor (one corrosion sensor sitting inside the plastic tank and one outside), a DC power supply, a digital multimeter, and two FBG temperature sensors. One of the FBG temperature sensors was used to measure the temperature of the electrolyte solution and one to measure the air temperature of the laboratory. The sacrificial steel specimen was attached to one of the corrosion detection sensors, and the other corrosion detection sensor was left in the zero strain state, sitting outside of the plastic tank. The sensor with the attached sacrificial steel specimen plus a temperature sensing FBG were submerged in the electrolyte, as shown in Figure 15. As Figure 15 shows, only the steel part was submerged. 

**Figure 16:**
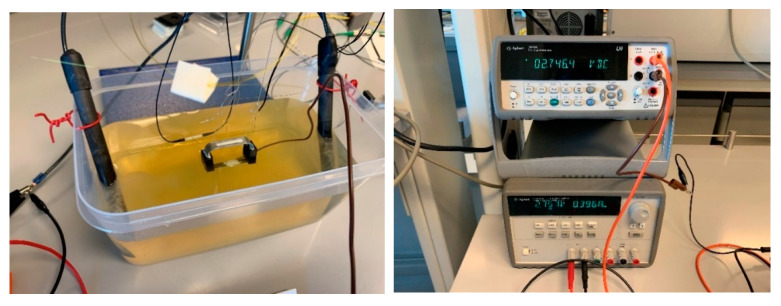
Start of the experiment.

**Figure 17:**
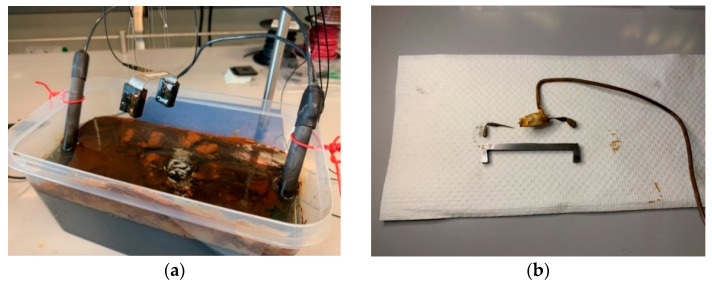
End of the experiment. (**a**) NaCl/water solution after corrosion. (**b**) Sacrificial Specimen before and after corrosion.

**Figure 18:**
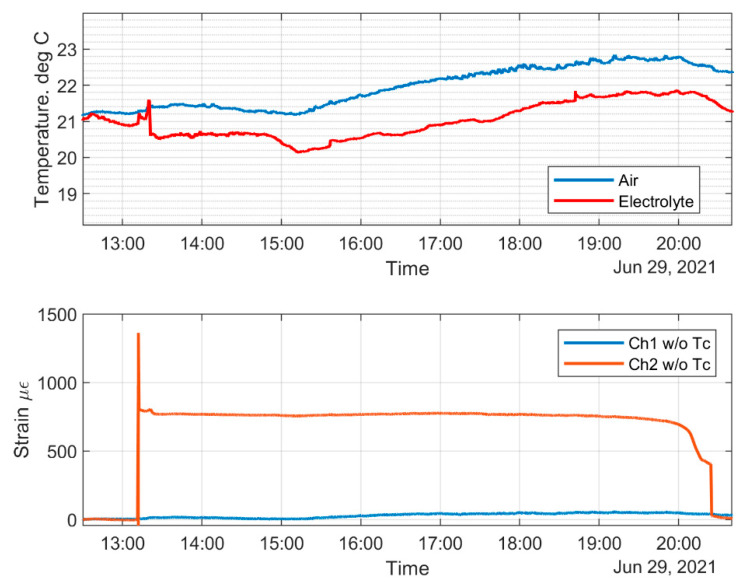
Top plot: air temperature versus electrolyte temperature. Bottom plot: strain measured on both corrosion detection sensors without temperature compensation.

The current was set to a low amperage, about 0.4 amps, to avoid heating of the electrolyte solution, and the DC voltage was set to 2.75 volts; see Figure 16. Once the electricity was turned on, hydrogen bubbles formed on the graphite electrodes, as shown in Figure 16. After 7 h, the sacrificial steel part was almost entirely corroded with a fairly uniform corrosion, with a higher corrosion rate near the cable attachment point; see Figure 17b. Figure 18 shows the temperature of the electrolyte solution versus the air temperature of the laboratory. It can be clearly seen that the temperature variations were small, within ±1 °C, and the solution temperature did not rise much throughout the 7 h experiment.

**Figure 19:**
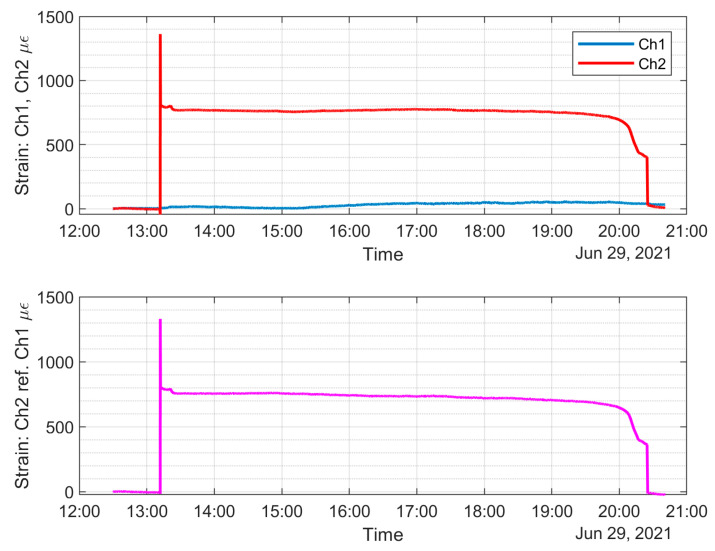
Top plot: strain on both FBGs without temperature compensation. Bottom plot: temperature-compensated strain on the corrosion detection sensor.

**Figure 20:**
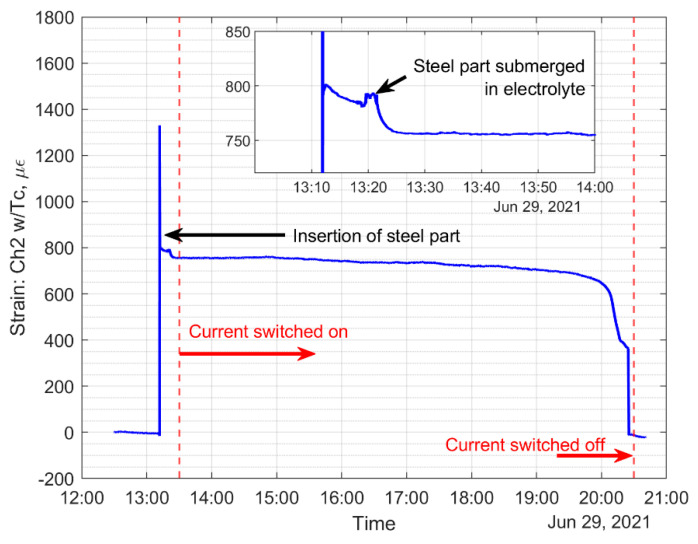
Timeline of the experiment, current switched on at 13:30, after strain stabilized from immersion into electrolyte.

The top plot of Figure 19 shows the strain on both FBG sensors without temperature compensation, and the bottom plot shows the temperature-compensated strain on the corrosion detection sensor, which is about 750 με. Figure 20 shows the timeline of the experiment, and the strain data show a gradual decrease as corrosion progresses. The gradual decrease of strain on the sacrificial steel specimen can be a sign of corrosion on the oil and gas pipeline, since the corrosion environment is very similar, and this gradual decrease in strain can be utilized to initiate an inspection of the pipeline at that location and possibly prevent further pipeline corrosion by implementing corrosion inhibition measures.

#### Analytical Results Versus Experimental Results for Design Concept #2

In design concept #2, when the steel specimen is inserted into the remaining part of the sensor, if the aluminum U-shaped part is properly manufactured, a *δ* motion of 0.71 mm is to be applied to the aluminum/plastic assembly at each end. As was indicated earlier, a 1-degree angle change in the 90-degree angle can change *δ* by ±0.3 mm. By the nature of the stamping, the possibility of the 90-degree angle being larger than 90 degrees is higher than being less than 90 degrees. Therefore, the results in Table 2 were calculated with *δ* ranging from 0.71 mm to 1.01 mm. In Table 2, the rows highlighted by the light gray color are the outputs.

Table 2 indicates that design Equations (13) and (20) did a very good job of predicting the experimental strain results. It is important to again mention that the exact value of the strain experienced by the U-shaped component is not critical, provided that the optical fiber strain is below its strain limit and the strain is significant enough to be detected by the optical interrogator.

### 6.2. Experimental Validation of Theory at Higher Temperatures

**Figure 21:**
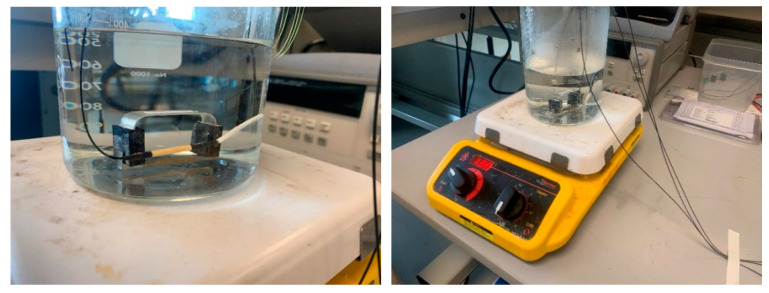
Corrosion sensor, given a temperature change, Δ*T*.

To see the effect of temperature on the strain of the corrosion detection sensor, validating the equations of Section 4.3, the following experiment was conducted. To give a uniform temperature change to the corrosion detection sensor of this paper (design concept #2), the sensor was placed in a beaker with water on a hot plate, as shown in Figure 21. An FBG temperature sensor was placed near the corrosion sensor in the same water solution, as shown in Figure 21.

**Figure 22:**
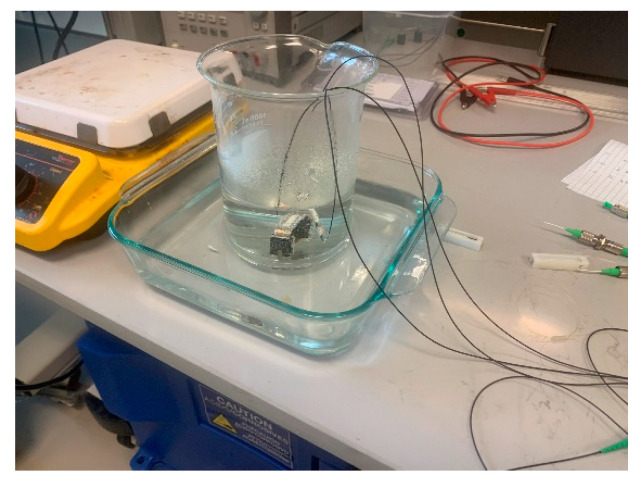
Room temperature water bath.

The water temperature was increased via the hot plate. At about 55 °C, the beaker with both sensors was removed from the hot plate and placed in a room temperature water bath, as shown in Figure 22.

**Figure 23:**
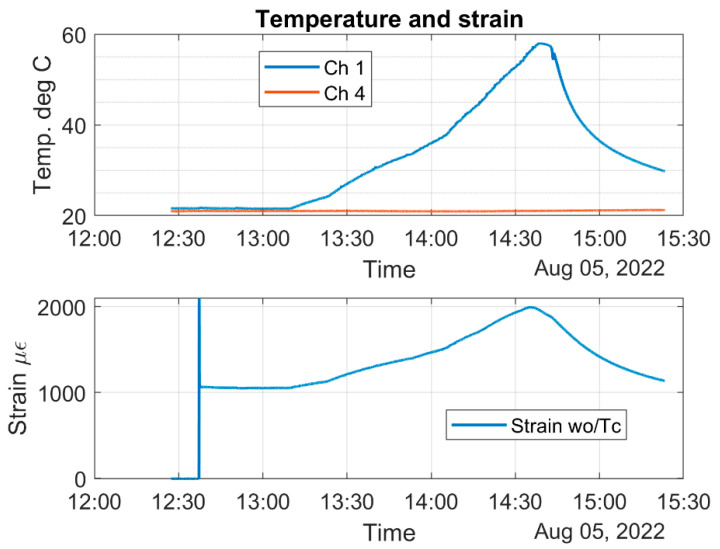
Temperature of the lab and the water solution and sensor’s strain versus time.

Figure 23 shows the laboratory temperature (labeled as Ch. 4, red color signal), water solution temperature (labeled as Ch1, blue color signal), and strain induced on the top surface of the aluminum component and the optical fiber versus time.

**Figure 24:**
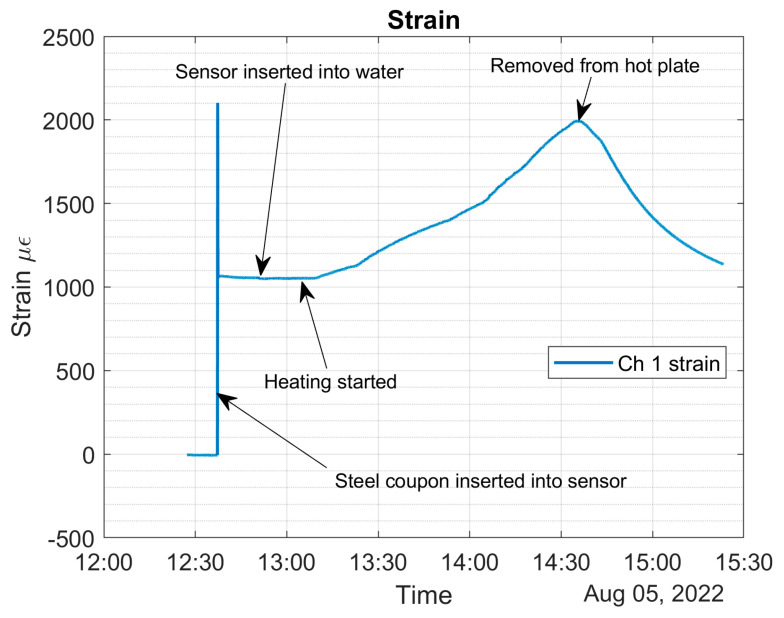
Corrosion sensor strain versus time in the heated water solution.

Figure 24 shows the strain of the corrosion sensor versus time. Figure 24 also shows when the heating started and when it stopped. It can be clearly seen that, before heating started, the mechanical strain, at room temperature, was about 1050 με. The highest strain registered was 2000 με. Notice that the room temperature strain (1050 με) of Figure 24 is higher than the room temperature strain of Figure 20 (750 με). For this experiment, we used a new corrosion sensor, and these data clearly show that, if the 90-degree angle of the aluminum U-shaped component is not maintained, higher values of the strain are to be expected.

**Figure 25:**
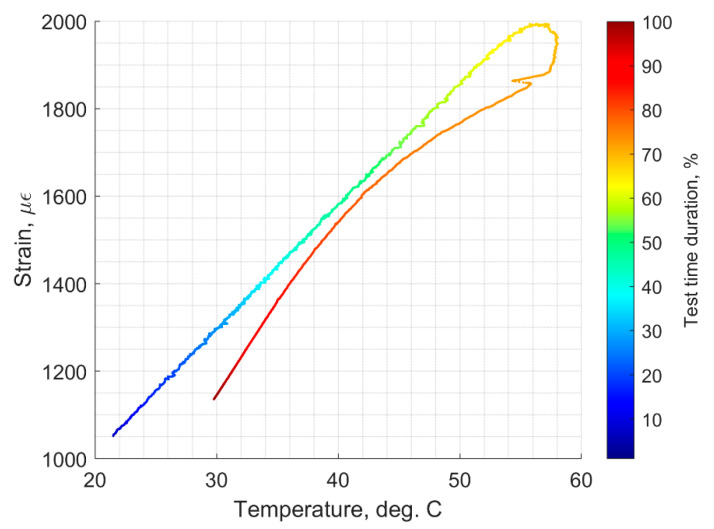
Corrosion sensor strain versus temperature.

Even though the heating of the water stopped at 55 °C, the water temperature rose and reached a temperature of 58 °C, as shown in Figure 25. The strain of 2000 με corresponds to the temperature of 58 °C, as shown in Figure 25.

From all the above graphs, it can be concluded that the temperature change is about 37 degrees Celsius (ΔT=58−21=37 °C), and the strain change due to temperature is about 950 με ($\varepsilon_{\Delta T}=2000-1050$). The epoxy that was used to bond the optical fiber to the U-shaped aluminum component is only rated for long-term exposure to 49 °C, and that is why we stopped heating the water at 55 °C. As can be seen from the above figure, during the heating, the sensor responds with a linear strain change up to about 50 °C. A slight creep was noticed, starting above 50 °C. We shall now verify Equation (25).

#### 6.2.1. Aluminum U-Shaped Component Subject to Δ*T* of 37 °C

The length of the aluminum U-shaped component, end to end, is 41.27 mm (see Figure 4). The experimental data showed the room temperature to be about 21 °C and the operating temperature to be 58 °C; thus, the change in temperature is:

ΔT=58−21=37  °C

The coefficient of thermal expansion for the aluminum component was assumed as follows:

$$\alpha_{Al}=0.24\times 10^{-4}\;\frac{m}{m\;{}^{\circ}C}$$

As a result of the 37 °C temperature change, the length of the aluminum U-shaped component will increase by:

$$\Delta L=\alpha_{Al}\Delta T\;L=\left(0.24E-4\right)\times 37\times 41.27=0.0366\;\mathrm{mm}$$

meaning each end of the components will elongate by $\delta_{Al}=\Delta L/2$, meaning 0.0183 mm due to the temperature change, Δ*T*. 

The strain induced on the top surface of the aluminum U-shaped component due to the temperature change will be:

$$\Delta \epsilon =\alpha_{Al}\Delta T=\left(0.24E-4\right)\times 37=0.000888\;\mathrm{or}\;888\;\mu m/m$$

#### 6.2.2. U-Shaped Sacrificial Steel Component Subject to Δ*T*

**Figure 26:**
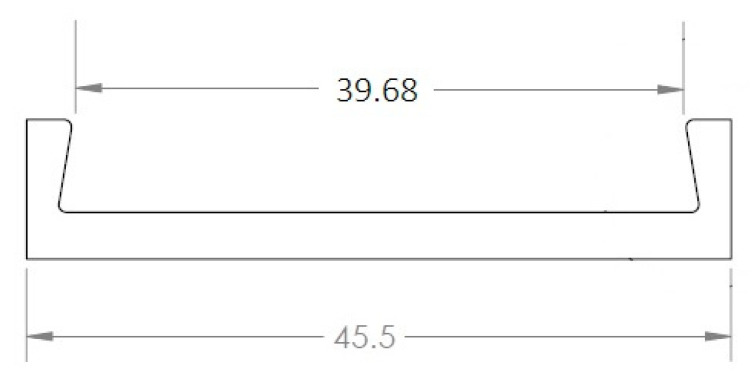
U-shaped sacrificial steel specimen dimensions.

The inner length of the U-shaped sacrificial steel specimen, as shown in Figure 26, is 39.68 mm.

The following coefficient of thermal expansion was assumed for the steel:

$$\alpha_{st}=0.122\times 10^{-4}\;\frac{m}{m\;{}^{\circ}C}$$

As a result of the 37 °C temperature change, the length of the U-shaped sacrificial steel specimen will increase by:

$$\Delta L=\alpha_{st}\Delta T\;L=\left(0.122E-4\right)\times 37\times 39.68=0.01791\;\mathrm{mm}$$

meaning both inner edges of the above component will elongate by ΔL2, meaning 0.00896 mm.

#### 6.2.3. Plastic Component Subject to Δ*T*

**Figure 27:**
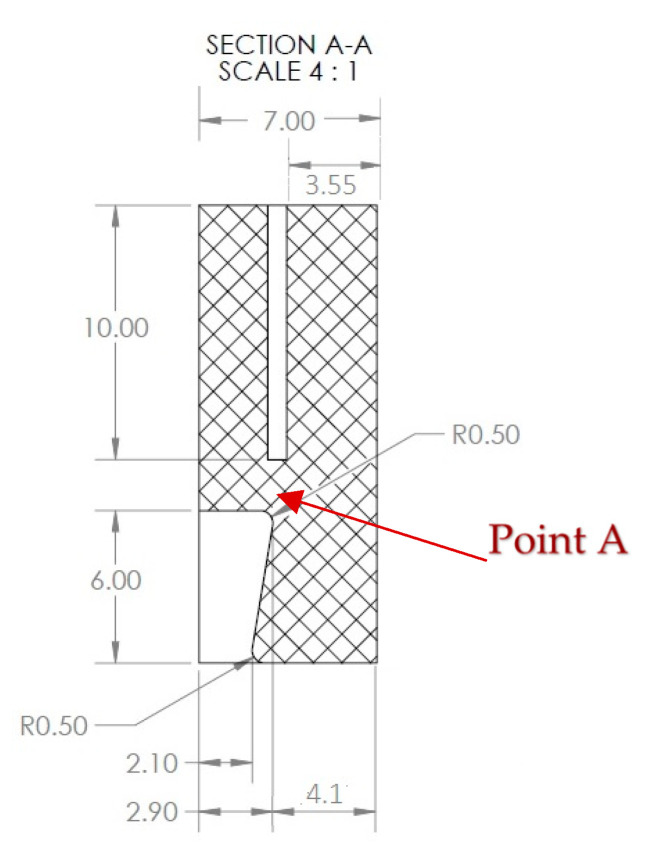
Plastic component 2D cross-section.

Since the U-shaped aluminum component is inserted into the slot of the plastic component, as shown in Figure 27, the motion of the slot will be similar to the motion of the U-shaped aluminum component. Here, we assumed the slot is fixed and saw how the plastic component expands due to the 37 °C temperature change.

The following coefficient of thermal expansion was assumed for the plastic:

$$\alpha_{pl}=0.31\times 10^{-4}\;\frac{m}{m\;{}^{\circ}C}$$

As a result of the 37 °C temperature change, Point A, relative to the right edge of the slot, will increase by:

$$\Delta L=\alpha_{Pl}\Delta T\;L=\left(0.31E-4\right)\times 37\times \left(4.1-3.55\right)=0.0006309\;\mathrm{mm}$$

meaning each end of the plastic component (Point A) will elongate by ΔL, meaning 0.0006309 mm.

#### 6.2.4. Overall Change in δ

The overall change in *δ* (in millimeter) will be:

$$\delta_{total}=\delta_{m}+\delta_{\Delta T-\alpha }=\delta_{m}+\delta_{Al}+\delta_{Pl}-\delta_{St}$$

$$\delta_{total}=\delta_{m}+0.0183+0.0006309-0.00896=\delta_{m}+0.00999$$

where the original mechanical deflection $\delta_{m}$ was in between 0.71 and 1.01 mm, so we can clearly see that $\delta_{\Delta T-\alpha }$ of 0.00999 mm is too small in comparison to the original mechanical deflection induced by the sacrificial steel specimen.

$$0.71999<\delta_{total}<1.01999$$

We now substituted all the calculated parameters into Equation (25) and calculated the overall strain experienced by the optical fiber. As explained earlier, our focus is on the last two terms of Equation (25). From the experiment (see Figure 24), we already know that the first term of Equation (25) is about 1050 με.

$$\epsilon_{total}=1050+\frac{\delta_{\Delta T-\alpha }\left(6y_{max}+6L_{1}-h\right)}{wh^{2}\left(\frac{L_{1}^{3}}{3I}+\frac{2\left(1+\mu \right)CL_{1}}{A}+\frac{L_{1}^{2}D}{2I}+\frac{D}{2A}\right)}+\alpha_{Al}\Delta T$$

Substitution of the parameters gives:

$$\epsilon_{total}=1050+7+888=1945\;\mu \varepsilon$$

The error between the experimental data (2000 με) and calculation (1945 με) is only 2.75%. The 2.75% error and experimental results clearly validate the accuracy of Equation (25).

#### 6.2.5. Optical Fiber Strain at 130 °C

In our application, the oil pipeline temperature is about 130 °C. At 130 °C, the strain experienced by the optical fiber and the aluminum component is calculated as follows:

$$\epsilon_{total}=1050+\frac{\delta_{\Delta T}\left(6y_{max}+6L_{1}-h\right)}{wh^{2}\left(\frac{L_{1}^{3}}{3I}+\frac{2\left(1+\mu \right)CL_{1}}{A}+\frac{L_{1}^{2}D}{2I}+\frac{D}{2A}\right)}+\alpha_{Al}\Delta T$$

Substitution of parameters and a Δ*T* of 109 degrees (ΔT=130−21=109) give:

$$\epsilon_{total}=1050+21+2616=3687\;\mu \varepsilon$$

The above calculations indicate that, in general, the optical fiber strain, induced as a result of the mismatch of the coefficient of thermal expansion of the three materials (namely, aluminum, plastic, and the steel), is much smaller than the strain induced by the thermal expansion of the aluminum due to Δ*T*. The dominant strains felt by the optical fiber will be due to the mechanical strain given to the aluminum component when the sacrificial steel specimen is attached plus the strain due to the temperature change of the aluminum component:

$$\epsilon_{total}\approx \;\epsilon_{m}+\alpha_{Al}\Delta T$$

## 7. Discussion

### 7.1. Field Implementation

**Figure 28:**
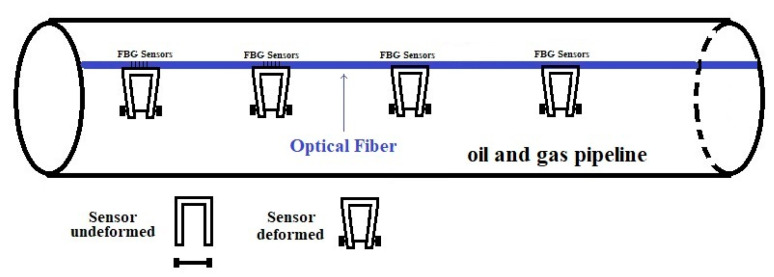
Corrosion detection sensor field implementation.

Figure 28 shows how the sensor design concepts of Figure 2 or Figure 3 would be implemented in the field.

The U-shaped plastic or aluminum component will be permanently glued to the FBG sensors of the optical fibers, as shown in Figure 28. Several optical fibers with FBG sensors will be used for each oil and gas pipeline. The bundle of the optical fibers will be attached to the oil and gas pipelines using zip ties. The dumbbell-shaped sacrificial steel specimen of design 1 (see Figure 1) or the U-shaped sacrificial steel specimen of design 2 (see Figure 5), made from the same oil and gas pipeline material, will be attached to the U-shaped plastic or aluminum component. The attachment of the sacrificial steel specimen to the U-shaped plastic or aluminum component places the FBG sensors in tension. The temperature of the pipeline creates additional strain on the plastic or aluminum U-shaped component. The corrosion detection sensors of this paper would not be attached or bonded to the oil or gas pipelines; thus, they will not experience the pipeline strains and stresses induced by the internal operating pressure or sand-dune or soil-induced loadings. In addition, there is no cover or protection for the corrosion detection sensors since it is desired to have the corrosion sensors experience the same corrosion environment as the pipelines.

When corrosion is serious at any pipeline location, the sacrificial steel specimen at that location corrodes and eventually fails and breaks into two pieces; thus, at that FBG sensor location, there will be a sudden drop in strain, since the mechanical tension strain is relieved. Once there is a strain drop on the interrogator display monitor at a particular pipeline location, the inspection of the pipeline at that FBG sensor location should take place. If the pipeline corrosion is severe, repairs of the pipeline will be conducted, and if it is not severe, ultrasound, X-ray, or eddy current inspection of that pipeline location will be conducted to find the severity of the pipeline corrosion. Once the pipeline is repaired or found acceptable for operation, the corroded sacrificial steel specimen will be replaced with a new one until the next corrosion failure. Since the dimension of the sacrificial steel specimen is known and the time to failure is also known, the average corrosion rate can be calculated.

Since, in oil fields, flammable liquids and gases are present, the signal transmission between the corrosion detection sensors and the control room must be spark-free. In this paper, optical fibers are the means of communication between the sensors and the control room. The optical interrogator needs to be placed in the control room since electricity is used to power the interrogators. Since light is the only communication mode between the sensors and the control room, and there are no batteries or electricity of any kind near the oil and gas pipelines, so the proposed corrosion detection system of this paper is very safe for oil fields. In addition, the sensor solution of this paper does not involve any holes or any design modifications to the oil and gas pipelines.

The number of FBG sensors that a single optical fiber can have depends on the wavelength range of the interrogator and the wavelength range given to each FBG sensor. The majority of interrogators on the market have a wavelength range of 60 nm to 80 nm. Some of the latest interrogators on the market now have a wavelength range of 100 nm to 160 nm. The commercially available 160 nm interrogator comes with 16 channels, and the 100 nm interrogator comes with 4 channels; however, with an optical switch, 64 independent channels can be obtained. FBG strain sensors are normally given a 4 nm range. With the 160 nm interrogator, a total of about 40 FBG sensors can be implemented on each optical fiber. With 16 channels, thus 16 optical fibers, each having 40 FBG sensors, a total of 640 FBG sensors can be placed on each pipeline. With the use of optical switches, we can further increase the number of FBG sensors, thus increasing the number of corrosion detection sensors on each pipeline. The corrosion status of the pipeline at any point, in between the corrosion detection sensors, can be predicted using the corrosion status of the two adjacent sensors.

### 7.2. Sensor Manufacturing and Field Implementation Cost

Since three- to four-thousand flowlines with lengths of 1 to 4 km may be found in a typical oil field and millions of corrosion sensors may be needed for corrosion detection, there is a genuine need to keep the cost of the corrosion sensors low. To keep the production cost of sensor design 2 low (see Figure 8), plastic molds can be used to manufacture the black-colored plastic components. As for the U-shaped aluminum component and the U-shaped sacrificial steel specimen, sheet metal heavy gauge stamping is an effective way to produce a large number of metal components for our proposed sensor at a low cost. This means the aluminum U-shaped component can be easily manufactured by metal sheet stamping. The design of the sacrificial steel specimen, made from API-5L-X65 steel, can be slightly modified in such a way that stamping can also be used to mass-produce that piece.

Since corrosion occurs slowly, there is no need to monitor the corrosion of each oil and gas pipeline in real-time. Since optical interrogators are expensive, the best way to keep the field implementation cost low is to use few interrogators, but to use more optical switches to direct the laser light from the interrogator to the bundle of fibers, placed on each pipeline. Quasi-real-time corrosion monitoring is perfectly sufficient for corrosion health monitoring of oil and gas pipelines since the external corrosion rate of oil and gas pipelines is about 0.4 mm per year [45].

## 8. Conclusions

A permanently field-deployed corrosion sensor for oil and gas flowlines was proposed. The corrosion sensor of this paper requires no power; it is very cost-effective, monitors corrosivity near the outer surface of the pipeline, and can provide an average corrosion rate over a period for oil and gas pipelines. While the sensor is not able to provide a direct measurement of the corrosion rate on the surface of the pipe, it shall be able to provide an estimate of the average corrosion rate at a given location based on the life of the sacrificial steel coupon. Since the pipe surface and the sensor are subjected to nearly the same environmental conditions, it is logical to presume that there will be a correlation between the corrosion rate on the pipe surface and that of the sacrificial specimen.

The pipelines that were the focus of this paper are small-diameter pipelines (4–8 inches), nearest the well, and the majority of these pipelines (called flowlines) are not coated or insulated (at least in the Middle East and many other locations with warm climates). Of course, the sensors developed here are “point” sensors, and they are not meant to provide an accurate measure of the wall thickness over the entire area of the pipe surface. Usually, some locations along the length of the pipe will corrode faster than others. The main purpose of these sensors is to respond to the corrosive environment near the pipe and help to determine the corrosivity hot spots along the pipeline.

The corrosion health monitoring system of this paper consists of (1) millions of corrosion detection sensors, each sensor consisting of a U-shaped plastic or aluminum component, and a sacrificial metal made from the same pipeline material under corrosion health monitoring, (2) a bundle of sixteen optical fibers per pipeline with up to 40 FBG sensors per optical fiber, (3) and interrogators and optical switches. The bundle of optical fibers is attached to the oil and gas pipelines using zip ties. The corrosion sensors are permanently bonded to the FBG sensors, but not to the pipeline’s outer surface. Attaching the sacrificial metals to the U-shaped components places the FBG sensors in tension. The fibers are either directly connected to an interrogator or via an optical switch to the interrogator. In oil fields, all electronics including the optical switches and interrogator(s), which can cause electrical sparks, need to be left in the control room. The optical fibers are the communication means between the corrosion sensors and the control room.

When the rate of corrosion is high at any given area on the pipeline, since the sacrificial steel specimen is also at the outer surface of the pipeline, it will very likely experience the same corrosion rate as the pipeline. We believe that the sacrificial coupon material will be exposed to the same (or very similar) environmental conditions (temperature, humidity, time of wetness, presence of certain chemical species) as the pipe surface. Hence, the degradation mechanism that the sensor will be subjected to will be largely similar to that of the pipe surface in the immediate vicinity of the sensor. At some point, the corrosion damage will be so large, that the sacrificial steel specimen will fail; thus, the strain of the corrosion detection sensor will have a sudden drop, and a change in strain signal will be observed at the interrogator in the control room. Once a signal is picked up in the control room, a visual inspection of the pipeline at that specific location can be conducted. 

If the pipeline corrosion is not severe, the sacrificial steel specimen is replaced with a new one until the next failure. If severe corrosion is found at any pipeline location, to access the depth of the corrosion damage, the pipeline can be inspected in detail using X-rays, ultrasonic thickness gauges, eddy current probes, or other inspection methods. Depending on the severity of the corrosion, the pipeline may be repaired, and the sacrificial steel specimen will be replaced until the next sensor failure.

The thickness of the sacrificial steel specimen can be chosen as needed, depending on the known average corrosion rate at any oil field. A default corrosion rate of 0.4 mm/year is proposed by the NACE RP-05020 standard [45]. If a 2 mm-thick sacrificial steel specimen is used, using the 0.4 mm/year corrosion rate, it would take 5 years or less for the sacrificial steel specimen to completely corrode through the 2 mm thickness. In most oil fields, ILI smart PIGs are generally used every three to seven years, to measure pipeline wall thickness. The thickness of the sacrificial steel specimen can be purposely chosen such that it can fail faster than the ILI smart PIG inspection interval.

Since the corrosion detection sensor of this paper will be in close proximity to the pipeline’s outer surface, its temperature will be very close to the pipeline surface temperature. The pipeline temperature and its temperature fluctuations can impact the strain reading and may confuse control room operators if corrosion damage has occurred or not. The best way to eliminate the sensor strain change due to temperature is to have an optical fiber with FBG temperature sensors at the same locations where the corrosion detection sensors are and subtract the effect of temperature-induced strain from the overall measured strain. If it is too costly to add additional optical fibers with FBG temperature sensors, then the control room operators can simply keep track of the sudden drop in strain, which never recovers, indicative of corrosion damage at any particular pipeline location.

## Figures and Tables

**Table 1:** Baseline parameters used in ANSYS to verify Equations (13) and (20).

Parameters	Parameter Description	Value
*h*	Sensor thickness	3 mm
*L*	Length of beams #1 and #3	20 mm
*D*	Length of beam #2	35 mm
*δ*	Displacement given to beams #1 and #3	0.65 mm
*L*_1_	Location where *δ* of 0.65 mm is applied	13.5 mm
*E*	Young’s modulus of the U-shaped plastic component	2.2 GPa
*μ*	Poisson’s ratio of the U-shaped plastic component	0.37
*ε_t_*_Eqn13_	Equation (13), calculated top surface strain of beam #2	4038 με
*ε_t_*_ANSYS_	Calculated top surface strain of beam #2 using ANSYS (Geom OFF)	4185 με
Error	Error between analytically calculated strain versus ANSYS-calculated strain (Geometric Nonlinearity is OFF)	3.6%
*ε_t_*_Eqn20_	Equation (20), calculated top surface strain of beam #2	4175 με
*ε_t_*_ANSYS_	Calculated top surface strain of beam #2 using ANSYS (Geom ON)	4306 με
Error	Error between analytically calculated strain versus ANSYS-calculated strain (geometric nonlinearity is ON)	3.1%

**Table 2:** Baseline parameters used to verify Equations (13) and (20).

Parameters	Parameter Description	Value
*h*	Sensor (aluminum) thickness	0.635 mm
*L*	Length of beams 1 and 3	20 mm
*D*	Length of beam 2	41.27 mm
*δ*	Displacement given to beams 1 and 3 (see Figure 9a)	0.71 to 1.01 mm
*L*_1_	Location where *δ* is applied (see Figure 9a)	17.315 mm
*Ε*	Young’s modulus of the aluminum U-shaped component	70 GPa
*μ*	Poisson’s ratio of the aluminum U-shaped component	0.3
*ε_t_*_Eqn13_	Equation (13), calculated top surface strain of beam #2	510 to 726 με
*ε_t_*_exp_	Experimental results	750 με
Error	Error between calculated strain versus experimental results	3.2% to 22.4%
*ε_t_*_Eqn20_	Equation (20), calculated top surface strain of beam #2	518 to 741 με
*ε_t_*_exp_	Experimental results	750 με
Error	Error between calculated strain versus experimental results	1.2 % to 21.1%

## Data Availability

The data presented in this study are available upon request from the corresponding author.
